# Genetic Factors and Their Role in the Pathogenesis of Biliary Atresia

**DOI:** 10.3389/fped.2022.912154

**Published:** 2022-06-29

**Authors:** Li-Na Wu, Zhi-Jun Zhu, Li-Ying Sun

**Affiliations:** ^1^Department of Critical Liver Diseases, Liver Research Center, Beijing Friendship Hospital, Capital Medical University, Beijing, China; ^2^Liver Transplantation Center, National Clinical Research Center for Digestive Diseases, Beijing Friendship Hospital, Capital Medical University, Beijing, China; ^3^Clinical Center for Pediatric Liver Transplantation, Capital Medical University, Beijing, China

**Keywords:** Biliary Atresia, gene, pathology, pathogenesis, Single Nucleotide Polymorphism

## Abstract

Biliary Atresia, a common basis for neonatal cholestasis and primary indication for Liver Transplantation, accounts for 60% of pediatric Liver Transplantations. While the pathogenesis of Biliary Atresia remains obscure, abnormalities within bile ducts and the liver, inflammation, fibrosis and cilia defects are thought to comprise the pathological basis for this condition. The findings of genetic variants in Biliary Atresia, such as Copy Number Variations and Single Nucleotide Polymorphism, are considered as essential factors in the development of this condition. In this review, we summarize and analyze these Biliary Atresia variants from a perspective of their pathological characteristics. In conclusion, such analyses may offer novel insights into the pathogenesis of Biliary Atresia and provide a foundation for future studies directed toward a better understanding and treatment of Biliary Atresia.

## Introduction

Neonatal cholestasis liver disease represents the principal liver disease in infants, with an incidence of 1 case in every 2,500 newborns (1). Biliary Atresia (BA) is a common basis for neonatal cholestasis and destructive inflammatory obliterative cholangiopathy in newborns, processes which can then affect both intra- and extra-hepatic bile ducts. The incidence of BA ranges from 1/12,000 to 18,000 in Western Europe (2), while higher rates are found in Asia, with 1.04 per 10,000 in Japan (3) and 3.7 per 10,000 in Taiwan (4). The symptoms of BA intially present as jaundice, acholic stools, dark urine and high levels of serum bilirubin. Children with BA failing to receive a Kasai porteoenterostomy (KPE) will progress to cirrhosis, liver failure and, the portal hypertension that can occur within 2 years following their birth will eventually necessitate a liver transplantation (LT) (5). Although KPE significantly improves early outcomes in children with BA, ~75% of these children will eventually require a LT (5). Histopathological features in liver specimens of BA include bile ductular proliferation (BDP), portal infiltrates, giant cells, hepatocellular swelling and fibrosis (6, 7). Depending on clinical phenotypes, BA is classified as perinatal (non-syndromic), embryonic, cystic and CMV-associated clinical variants (8).

Currently, the exact pathogenesis of BA remains unclear. Most investigators working in this area believe that BA is not a disease with a single etiology but a combination of different phenotypes that share certain clinical features, such as obliteration of the biliary tree early in life. The etiology of BA includes effects resulting from viral perinatal infections, toxins, immunity dysregulation and genetic mutations (5, 9, 10). The existence of multiple viruses (rotavirus, CMV, EBV) in injured livers and biliary remnants or serological markers in serum of BA patients (11) and rotavirus-induced murine models of BA have revealed an important role for these viruses in the pathogenesis of BA (8, 12). In addition, it has been reported that an interaction of gut microbiota with dysmetabolism of tryptophan and bile acid may aggravate the liver damage resulting from BA (13). The discovery of Biliatresone demonstrates toxins may be necessary for the pathogenesis of BA. Waisbourd-Zinman showed that Biliatresone disrupted the extrahepatic biliary tree and subepithelial fibrosis by reducing Glutathione and SOX17 (14). In the development of BA, the immune system plays a role in injuries of bile tracts (15), and an increased expression of lymphocyte activation (LFA-1, IL-2 receptor) and proliferation (transferrin receptor) were found to be present in the portal vein of patients with BA. Results from a recent report have suggested that BA may involve an autoimmune injury of the bile duct resulting from viral infections (16). It is also widely believed that genetic factors play an important role, as several genes have been identified to be associated with BA, including *ADD3, EFEMP1, MMP7*, and *ARF6*. With the development of GWAS, a number of Copy Number Variations (CNVs) and Single Nucleotide Polymorphisms (SNPs) have been observed in BA and was highly associated with the pathogenesis of BA. Concluded by “The common-disease common-variant (CDCV) hypothesis”, variants which the Minor allele frequency (MAF) is more than 0.05 are usually thought to be common vatiants (17). A majority of these loci have been tested and verified in follow-up replication studies in different cohorts, with the result that multiple loci with small biological effects acting in various combinations appearing to contribute to the constellation of “minor” or “major” malformations associated with BA. In addition, other functional non-coding variants within gene regulatory elements may potentially result in a disease phenotype through modulating gene expression levels.

In this review, we summarize the genetic factors associated with BA and analyze the specific mechanisms of these as related to the pathogenesis of this condition.

## Genetic Factors in BA

**Figure 1:**
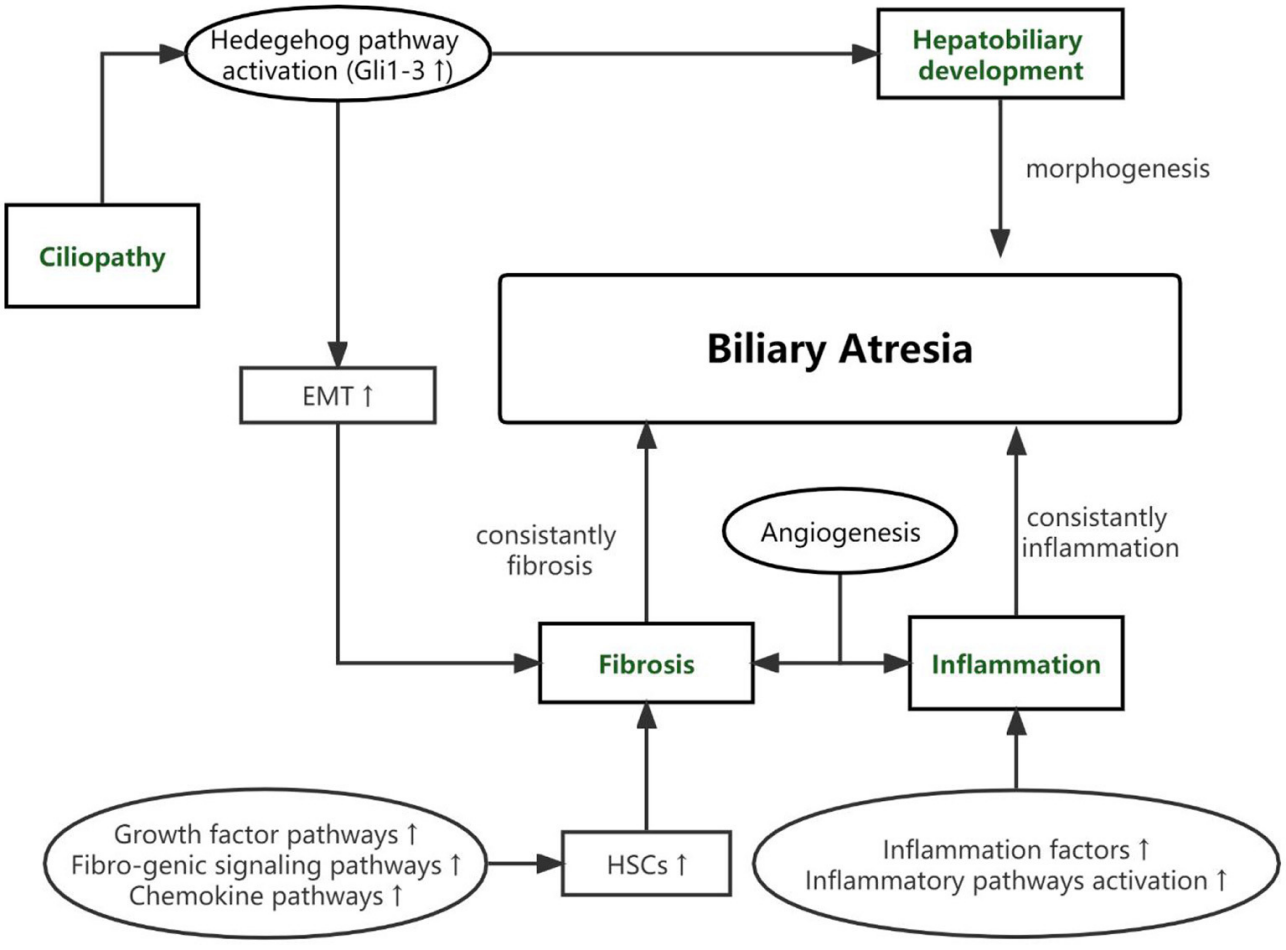
The connection among four pathways and between the pathogenic pathways of BA.

As based on the pathological features of BA and gene's function, potential genetic variants of BA can be classified into 4 pathways as presented in Table 1. The connection among four pathways and between the pathogenic pathways of BA is shown in Figure 1.

**Table 1:** Gene variants in BA and their potential role in the pathogenesis of BA.

**Gene**	**Location**	**Ethnic group**	**Variants**		**LD locus**	**Alleles**	**MA**	**MAF**	**Hepatobiliary development**	**Fibrosis**	**Inflammation**	**Ciliopathy**	**References**
			**Type**	**Func annot**									
*ADD3*	10q25.1-q25.2	Chinese	rs17095355	Intronic	rs2501577	C/T	T	0.494–0.551	X	X	-	-	(18, 19)
*GPC1*	2q27.3	Caucasian	CNV(deletion)	-	-	-	-	-	X	-	X	-	(20, 21)
		Chinese	rs2292832	Intronic	-	T/C	C	0.223					(22)
			rs3828336	Intronic	-	C/T	T	0.06					
		Chinese	rs6707262	5′upstream	rs6750380	T/C	C	0.438					(19)
			rs6750380	5′upstream	rs6707262	T/C	C	0.434					
*ARF6*	14q21.3	Caucasian	rs3126184	3′-UTR	rs10140366	T/C	T	0.2857	X	-	-	-	(23)
*MIR499*	20q11.22	Chinese	rs3746444	Intronic	NM	A/G	G	0.258	-	X	X	-	(24)
		Egyptian			NM			0.46					(25)
*ITGB2*	21q22.3	Chinese	rs1160263	3′-UTR	NM	G/T	T	0.191	-	-	X	-	(26)
*ICAM1*	19p13.2	Turkish	rs1799969	Missense	-	G/A	A	0.632	-	-	X	-	(27)
*MIF*	22q11.23	Turkish	rs755622	None	NM	G/C	C	0.611	-	X	X	-	(28)
		Egyptian	rs5844572	None	NM	insATTC	-	-					(29)
*VEGFA*	6p12	Chinese	rs3025039	3′-UTR	rs10434	C/T	T	0.089–0.154	-	X	X	-	(30, 31)
*PDGFA*	7p22	Chinese	rs9690350	Intronic	NM	G/C	C	0.37	-	X	X	-	(32)
*EFEMP1*	2p16.1	Chinese, European	rs10865291	Intronic	rs6761893, rs727878	G/A	A	0.43	-	X	-	-	(33)
*CD14*	5q31.3	Chinese	rs2569190	Intronic	NM	T/C	C	0.03	X	-	X	-	(34)
*NOTCH2*	1p12	Chinese	rs835576	3′ UTR	NM	A/G	G	0.42	X	-	-	-	(34)
*ADIPOQ*	3q27.3	Thai	rs1501299	Intronic	-	G/T	T	0.189	X	-	X	-	(35)
IL18^*^	11q22.2-q22.3	Chinese	rs187238	Promoter	rs549908	C/G	G	0.14074	-	-	X	-	(36)
			rs1946518	Promoter	-	T/G	G	0.408969	-	-	X	-	(36)
*ABCB11*	2q31.1	Vietnamese	rs2287622	Missense	NM	A/G	A	0.455	X	-	-	-	(37)
PKD1L1^*^	7p12.3	Multiracial	rs139293796	Exonic	NM	G/A	A	0.00275	X	-	-	X	(38)
			rs148011149			A/G	G	0.000288					
			rs776420484			A/G	G	3.00E-04					
			rs139858574			G/A	A	0.000308					
			rs528302390			AG/-	-	0.000912					
			rs143005953			G/A	A	0.006978					
			rs140456142			G/A	A	0.001154					
			rs770832954			C/T	T	4.00E-05					
			rs752673990			G/C	C	4.00E-05					
*PKHD1*	6p12.3-p12.2	Chinese	rs137852950	Missense	NM	A/C	C	1.00E-04	-	-	-	X	(39)
			rs139127465	Missense	NM	A/G	G	1.90E-04	-	-	-	X	(39)

*Alleles means REF/ALT, information from NCBI, all alleles are reported in the Forward orientation; NM, means not-mention*.

* *MAF from NCBI*.

We have collected articles related to biliary atresia and genes from 2005 to December 2021. Susceptible genes are selected according to the following criteria: (1) SNPs are significantly correlated with BA cohort; (2) It is verified by multiple cohorts or animal disease models; (3) its biological function is closely related to the four aspects described in our review. Some genes, such as *IL18, CD14, NOTCH2* and so on, are only reported by one cohort are not in text but in Table 1 (34–37, 40). Genes that may be susceptible genes but have not yet been found BA are selected according to the following criteria: (1) Abnormal expression in BA specimens; (2) Demonstrated by animal disease models; (3) Their biological function is closely related to the four aspects described in our review. This part of genes are collated into Table 2 (42, 44–47).

**Table 2:** Differential expressed gene in BA.

**Gene**	**Location**	**Function**	**References**
*HMGB1*	13q12.3	Inflammation	(41 )
*CTGF*	6q23.2	Fibrosis	(42)
*MMP7*	11q22.2		(43)
*MAN1A2*	1p12	Ciliapathy	(44)
*GATA6*	18q11.2	Hepatobiliary development	(45)

There also exist several potential-associated susceptibility loci that are listed in this review, although their validation await data from future studies with independent cohorts.

### BA and Hepatobiliary Development

As noted above, BA can be classified into either non-syndromic or syndromic BA. In newborns with syndromic BA, abnormal morphogenesis of biliary ducts begins early *in utero*, likely during the embryogenesis period (48). An uncommon type of BA, cystic biliary atresia (CBA), also demonstrates an abnormal morphogenesis as a pathological characteristic of BA (49). However, such effects, as associated with non-syndromic BA, remain unclear. Overall, extrahepatic biliary tract injury and fibrous obliteration continue to represent the fundamental injuries in BA (50, 51).

#### ADD3 and XPNPEP1

In 2010 Garcia-Barcelo et al. reported on 324 BA cases and 516 (481 after quality control) controls in a Chinese cohort study and were the first to show that SNP (rs17095355), was located between *ADD3* and *XPNPEP1* and was significantly correlated with BA. Linkage disequilibrium (LD) analysis showed that SNP rs17095355 and rs2501577 were in strong LD (*r*^2^ > 0.8) which means they nearly represent a same signal (18). *XPNPEP1* (X-Prolyl Aminopeptidase 1), which is located in 10q25.1 and encodes XPNPEP1, contributes to the degradation of bradykinin and substance P (52). Such degradation products participate in inflammation responses within many organs and diseases. *ADD3* (Adducin 3), which is located in 10q25.1-q25.2, and encodes Gamma-adducin, promotes cell-cell connections and adhesions in hepatocytes and biliary epithelial cells, regulates cell migration and adhesion and participates in contraction of bile cells as a membrane-cytoskeleton-associated protein (53). *XPNPEP1* has been shown to be expressed in all tissues so far examined, including pancreas, heart, muscle, kidney, liver, lung and brain (54). Tsai et al. found that although *XPNPEP1* showed an increased expression in the intra- and extra-hepatobiliary tracts in BA livers of a Caucasian cohort, no statistically significant differences were observed between the BA and two control (normal control and diseased control) groups, while *ADD3* demonstrated significantly higher expression levels in BA livers (55). These investigators also noted that the high degree of significance of rs7099604 in *ADD3* in this Caucasian cohort was attributable to regional differences in haplotype structure. In 2016, Tang et al. used a knockdown of *ADD3a* as a means to investigate the pathogenesis of BA in Zebrafish. Their results indicated that knockdown of *ADD3* induced intrahepatic defects and decreased biliary function while no such effects were observed in response to a knockdown of *XPNPEP1*. Similar results were observed in homozygous *ADD3a* mutants, and it was also suggested *ADD3* may lead to BA by affecting the Hedgehog pathway (56). Hedgehog (Hh) is one of a number of signaling pathways that is repeatedly used for intercellular communication in development (57) and plays a critical role in embryonic morphogenesis (58). Back in 2011, there were reports indicating that BA was associated with excessive Hedgehog activity, effects which can then stimulate mesenchymal transition in the bile duct epithelium (EMT) and lead to bile duct malformation. They demonstrated that the Hh-responsive nuclear transcription proteins, Gli1-3, were expressed significantly in the livers of BA patients, which means a highly increased activity of Hh signaling in BA. And the Gli-3 is correlated considerably with EMT, which means Hh signaling takes part in the fibrosis process in BA (59). Taken together, the studies described above provide strong evidence for the involvement of *ADD3* in the pathogenesis of BA.

#### GPC1

In 2010, Spinner et al. first reported the observation of overlapping heterozygous deletions at 2q27.3, the location of *GPC1*, in two unreleted BA patients (20). *GPC1* encodes glypican 1, a protein that modulates various signaling pathways including fibroblast growth factors (FGF), vascular endothelial growth factor A (*VEGFA*), transforming growth factor β (TGF-β), WNT and Hedgehog to modulate inflammatory responses in intercellular signaling (60). In a CNV study with 61 BA cases and 5,088 controls in an American cohort, Cui et al. observed a statistically significant increase in deletions at 2q37.3 in patients with BA that resulted in deletion of one copy of *GPC1*. Moreover, results as obtained by inducing *GPC1* defects in zebrafish, revealed that the abnormal morphogenesis in intra- and extra-hepatic bile ducts of these zebrafish embryos led to reduced gallbladder uptake, while suppressing the Hedgehog pathway partially reversed this biliary tract defect in these *GPC1* mutants. s deletion (21). Such findings strongly implicate *GPC1* as being involved in the pathogenesis of BA *via* the Hh signaling pathway. Ke et al. proposed that it may be the joint effects of gene-gene interactions that ultimately exert a substantial impact on the risk for BA (61). In 2016, Tang et al. demonstrated that a co-downregulation, but not an individual downregulation, of *ADD3* and *GPC1* produced a decline in biliary uptake function and biliary defects (56). These results support the proposal that these 2 genes may synergistically function within the Hedgehog pathway to mediate biliary defects. The variants of *GPC1* in BA are not just CNVs. In 2016, results from a study performed with 134 BA patients and 618 controls in a Chinese cohort indicated that rs2292832 has significantly relevance to the risk of BA, while rs3828336 appears to exert only marginal effects upon BA (22). Moreover, in 2020, Bai et al. found two SNPs (rs6707262 and rs6750380) in the *GPC1* gene that were significantly associated with BA in another Chinese cohort (19). These two SNPs were in nearly perfect LD (*r*^2^ = 0.98). In this way, through its capacity to induce abnormal morphogenesis of biliary tracts, *GPC1* plays an important role in the pathogenesis of BA.

#### ARF6

It was first proposed by Ningappa et al. that low expressions of induced *ARF6* (ADP Ribosylation Factor 6) result in a diminutive liver and comparatively sparse intrahepatic biliary tracts (62). Moreover, the expression of this gene is decreased in BA specimens. This gene lies on the 14q21.3, encodes ARF6 and, as a GTP-binding protein, is involved with protein trafficking that regulates cytoskeleton remodeling (52, 63). It has also been proposed that *ARF6* participates in the development of fetal hepatocytes. Following knockdown of the *ARF6* gene in embryos there is an abnormal formation of the hepatic cord (64). The findings that two SNPs, rs3126184 and rs10140366, map to the 8.763 kb upstream ARF6 gene and in linkage disequilibrium (*r*^2^ = 0.99) with each other (23), are positively correlated with BA provides further evidence for *ARF6* as being a susceptibility locus in BA. In summary, results from these studies demonstrate that an abnormal morphogenesis of biliary tracts, as can occur in response to low expressions of *ARF6*, may contribute to the basis for BA.

### BA and Inflammation

BA is a progressive fibro-inflammatory disease characterized by an overexpression of inflammatory factors. Within the liver and circulation of patients with BA, there is an increase in soluble inflammatory adhesion molecules and cytokines, which may serve as predictors of native liver survival after KPE (65, 66). However, even with KPE, the native liver may continue to experience consistent inflammation leading to liver cirrhosis. Genes associated with various aspects of the inflammatory pathway have been shown to exhibit variable polymorphism frequencies in patients with BA, thus suggesting that upregulation of proinflammatory genes might play an important role in BA pathogenesis.

#### MIR499

The first indication that the SNP (rs3746444) in *MIR499* (MicroRNA-499) was of relevance to BA and correlates with the increased expressions of inflammatory factors (IL-6, TNF-α) in the serum and liver of BA patients was presented in 2016 by Shan et al. The G allele of rs3746444 was related with the degree of inflammation. This polymorphism map to the stem region opposite the mature miR-499 and results in a mismatch, which might affect *MIR499* function. The LD information of rs3746444 is not mentioned in this article (24). These findings were corroborated in a study with an Egyptian cohort (100 BA cases, 100 cases with cholestatic liver diseases other than BA and 100 healthy controls) as conducted by Gawish et al. (25). This group also provided results indicating that rs3746444 was correlated with the degree of fibrosis and outcome after KPE in BA patients. The *MIR499* gene, located in 20q11.22, encodes micro RNAs which serve as a type of noncoding RNA involved in post-transcriptional regulation of gene expression in multicellular organisms, mainly involved with affecting the stability and translation of mRNA. These miRNAs lay a solid foundation for the generation and function of liver and bile ducts (67). This capacity for *MIR499* to promote inflammation and affect the generation and function of liver and bile ducts suggests that it plays an important role in the pathogenesis of BA.

#### ITGB2 and ICAM1

In 2013, Zheng et al. presented the first evidence that the SNP (rs1160263) in *ITGB2* (Integrin Subunit Beta 2) was strongly associated with BA pathogenesis. They also found *ITGB2* was overexpressed in BA liver through liver biopsy (26). Located in 21q22.3, this gene encodes an integrin beta chain, Integrinβ2 (CD18), which combines with a number of diverse alpha chains to generate different integrin heterodimers and is expressed in a variety of leukocytes, participates in leukocyte adhesion and migration and supports macrophage apoptosis and neutrophil phagocytosis (68, 69). Integrinβ2 (CD18), which is the subunit of lymphocyte-associated antigen-1(LFA-1) and macrophage-1(MAC-1), mediates the high affinity activity in a variety of cells that express intercellular adhesion molecules (ICAM, especially ICAM-1). Of particular relevance to this review were the findings presented almost 30 years ago demonstrating that *ICAM1* is overexpressed in extra- and intra-hepatic bile ducts of BA patients (70, 71) and, in 2008, Arikan et al. reported that the A allele of SNP (rs1799969) in *ICAM1* was positively correlated with BA (27). *ICAM1* is located in 19p13.2 and is a coding gene which encodes a cell surface glycoprotein (ICAM1/CD56) which is typically expressed in endothelial and immune system cells (52). In this way, *ICAM1* mainly participates in immunological and inflammatory signaling pathways including interferon and interleukin signaling (72). With regard to *ITGB2*, not only do *ITGB2* mediated inflammatory cells (mainly include T cell and Microphage cell) connect with cholangiocytes, but they also transmit co-stimulation signals during MAC-1 and LFA-1 antigen presentation. Given the above descriptions of their actions, both *ITGB2* and *ICAM1* genes would seem to be good candidate loci for genetic susceptibility to BA.

#### MIF

It was proposed in 2006 that C allele of rs755622 (*MIF*-173G/C polymorphism) in the *MIF* gene may correlate with the susceptibility for BA as revealed in a Turkish cohort study (28). The *MIF* gene encodes Macrophage Migration Inhibitory Factor (MIF) which serves as a proinflammatory factor that participates in autoimmunity in response to pathogens. Compared to normal controls MIF, as a proinflammatory factor, was elevated in BA patients (73). However, in a 2017 Egyptian cohort study, no correlations were found between the rs755622 and rs5844572 polymorphisms and susceptibility for BA, while it appears that rs5844572 may correlate with the degree of fibrosis and rate of BA progression (29). These differences in results may, in part, be attributable to the different populations studied. Further studies will be required to evaluate the role of *MIF* in BA pathogenesis.

### BA and Liver Fibrosis

Regardless of underlying etiology, angiogenesis represents a fundamental feature of chronic liver diseases. Angiogenesis is thought to promote fibrosis, primarily based upon the observation that changes in angiogenesis and fibrosis are simultaneously present within many organs, including the liver. Not only does angiogenesis promote liver fibrosis, but it also channels more nutrients to the bile tract to stimulate ductular proliferation. Fibrosis is a main characteristic of liver BA. Like inflammation, fibrosis can also persist within the native liver in BA, even after KPE, and this liver fibrosis has the potential to progress into cirrhosis. Activation of hepatic stellate cells (HSCs) represents the main process underlying liver fibrosis. In addition to HSCs, results from a number of studies have demonstrated that Epithelial Mesenchymal Transformation (EMT) also contributes to liver fibrosis *via* generation of collagen-producing myofibroblasts (74). It is possible that genetic polymorphisms may exist which can then lead to the angiogenesis and fibrosis associated with liver BA.

#### VEGFA

As observed in a sample of Han Chinese, *VEGFA* (Vascular Endothelial Growth Factor A) was found to be over-expressed in livers of BA patients and rs3025039 in *VEGFA* is involved in the development of BA (30). These findings were supported from results as obtained in a different cohort of Chinese *via* a follow-up replication study of this locus as reported by Liu et al. Liu et al. also found two-SNP LD block (rs3025039-rs10434, *r*^2^ > 0.8) significantly correlated with BA risk (global *P* < 0.001) by Haplotype analyses (31). This gene locates in 6p12 and encodes VEGFA which participates in angiogenesis and mediates vascular permeability, as well as participating in inflammation through the promotion of migration and induction of the activation of monocytes (52). Moreover, VEGFA release by cholangiocytes can act as a signal linking ductal and arterial development in the liver and thus promote arterial and peribiliary plexus angiogenesis (75). Most notably, the *VEGFA* rs3025039 polymorphism, in particular the C allele, is associated with BA and may possibly confer an increased degree of susceptibility for this disease. Accordingly, through its capacity to promote angiogenesis and inflammation, VEGFA is thought to be a susceptibility gene for BA.

#### PDGFA

Since 1995 it has been known that inflammation and fibrosis are correlated with TGF-β1 and PDGF levels in liver BA (76). In 2020, Liu et al. demonstrated that the G allele in rs9690350 of *PDGFA* (platelet-derived growth factor subunit) markedly increased the susceptibility for BA in newborns (32). *PDGFA* is located in 7p22 and encodes PDGFA. This growth factor plays an essential role in the regulation of embryonic development, cell proliferation, cell migration, survival and chemotaxis (52). In 2019, Yang et al. reported that PDGFR is overexpressed in fibrosis, as demonstrated in a mouse liver, and is positively correlated with fibrotic and angiogenic markers (77). This view was advanced with studies that involved inhibiting activation of the PDGF/PDGFR and TGFβ/Smad signaling pathways as performed in 2020 by Chen et al. who demonstrated that exogenous Thymosin4 could relieve cholestatic liver fibrosis in a bile duct ligation (BDL) mouse model (78). In this way, through the promotion of inflammation and fibrosis, *PDGFA* can participate in mechanisms contributing to BA.

#### EFEMP1

In 2018, Chen et al. presented the first evidence indicating that the *EFEMP1* (EGF-containing fibulin-like extracellular matrix protein 1) gene may be a potential gene in BA susceptibility as based on a cohort study including 343 BA cases and 1,716 healthy controls. They found that expression of this gene was markedly increased in BA patients, and three SNPs (rs10865291, rs6761893, and rs727878, the *r*^2^ between these are >0.5) were highly correlated with the occurrence of BA, results which suggest genome-wide significance (33). *EFEMP1* is located in 2p16.1 and encodes a member of the fibulin family of extracellular matrix glycoproteins (EFEMP1). This protein is involved with extracellular matrix, tissue regeneration, organogenesis and may play a role in cell adhesion and migration (52). It also binds EGFR, the EGF receptor, which then induces EGFR autophosphorylation and activation of downstream signaling pathways (79, 80). Chen et al. reported that the *EFEMP1* gene is highly expressed in extra cholangiocytes and vascular smooth muscle cells in BA as well as in other cholestatic diseases, while it is only expressed in vascular smooth muscle cells in healthy controls. It has also been shown that *EFEMP1* is over-expressed in portal vein fibroblasts in a rat model. Collectively, these results imply that *EFEMP1* may play a role in the liver fibrosis associated with BA.

### BA and Abnormal Cilia Formation

Primary cilia represent an evolutionarily conserved subcellular structure that exists in most cell types within the human body. Primary cilia coordinate a variety of signaling pathways, including those regulated by Hh, G protein-coupled receptors (GPCRs), WNT, receptor tyrosine kinases (RTKs) and TGFβ/bone morphogenetic protein (BMP), factors which are involved with the control of developmental processes, tissue plasticity and organ functions (57, 81). By sensing changes in the extracellular environment, primary cilia co-ordinate subsequent cascades of amplified signals throughout the cell. In 2012, Chu et al. reported that, as compared with that of healthy patients and other cholestasis liver diseases, primary cilia were found to be much shorter, less abundant and displayed abnormal orientations within the livers of BA patients (82). Following RRV infection, primary cilia were found to be selectively decreased within extrahepatic cholangiocytes of newborn mice as well as in BA specimens and in RRV-infected primary cholangiocytes and extrahepatic ducts as reported by Wells et al. (83). In 2021, Lam et al. detected some gene vatiants which is rare, deleterious *de novo* or biallelic variants in ciliary genes in non-syndromic BA patients, including *KIF3B, TTC17* and *PCNT* and so on (39). They found that knockdown of these genes in human cell and zebrafish models could resulted in intrinsic ciliary defects. The MAF of these gene variants <0.005. All of these results suggest that ciliary gene mutations can contribute to the development of BA phenotypes.

#### MAN1A2

In 2020 So et al. reported that knockdown of *MAN1A2* (Mannosidase Alpha Class 1A Member 2) results in poor biliary network formation, ciliary dysgenesis in Kupffer's vesicles, a dysregulation in the expressions of EGF, TGF and the Hedgehog pathway along with a decreased expression of genes involved with cilia development (44). This gene, which is located in 1p12, encodes MAN1A2 and is mainly expressed in the placenta and testes (84). These investigators also found that a simultaneous knockdown of both the *MAN1A2* and *ARF6* genes exerted a synergistic influence leading to significant abnormalities in the development of the bile ducts, while no such effects were observed with knockout of either gene alone (44). As noted above, *ARF6* was identified as a susceptibility gene for BA and now, these findings with *MAN1A2*, suggest that *MAN1A2* can affect ciliary development and EGFR signaling and regulate the formation of intrahepatic biliary networks by interacting with *ARF6*. However, to date, no variants in this gene have been found.

#### PKHD1

The possibility that *PKHD1* may participate in BA cilia dysplasia was initially propose by Hartley et al. (85). *PKHD1* (PKHD1 Ciliary IPT Domain Containing Fibrocystin/Polyductin) lies on 6p12.3-p12.2 and encodes fibrocystin which may act in collecting-duct and biliary differentiation as well as participate in the regulation of cholangiocytes proliferation and *CCN2* expression in a CXCL8-dependent manner (86, 87). Variants in this gene have been associated with a severe form of polycystic kidney disease and, in some cases, hepatic biliary tracts. This ciliopathy is characterized by dilatation of the collecting ducts and biliary fibrosis due to a ductal plate malformation. Ductal plate malformations are also a recognized feature of BA as based on histological examination, which suggests a similar pathogenesis to other ciliopathies (88–90). In 2021, an whole exome sequencing study in Chinese cohort demonstrated that there are two rare variants (rs137852950 and rs139127465) in *PKHD1* (39). Although no variants have been found so far, *PKHD1* may play a role in pathogenesis of BA through its capacity to affect cilia development.

#### PKD1L1

In 2019, Berauer et al. found 9 rare variants (rs139293796, rs148011149, rs776420484, rs139858574, rs528302390, rs143005953, rs140456142, rs770832954, rs752673990) in *PKD1L1* gene with BASM (Biliary Atresia Splenic Malformation Syndrome) patiets (38). The MAF of these variants are <0.05. They also demonstrated that *PKD1L1* is strongly expressed in cholangiocytes compared with liver tissue. *PKD1L1*, located in 7p12.3, encodes Polycystic kidney disease protein 1-like 1(PKD1L1) which is the component of a ciliary calcium channel and forms a heterodimer with PKD2L1 in primary cilia and forms a calcium-permeant ciliary channel that regulates sonic hedgehog/SHH signaling (91). Through affect the ciliopathies, hepatobiliary development, and cholestasis, *PKD1L1* is considered as a new candidate gene for BA.

## Discussion

As cholestasis and destructive inflammatory obliterative cholangiopathy neonatal event, BA has severe outcomes in newborns. However, pathogenesis has not been detected clearly. BA is not an isolated disease but a common pathology of various pathogenetic pathways. In this review, we divided BA through the function of susceptibility genes into abnormalities within bile ducts and the liver, inflammation, fibrosis, and cilia defects.

During the embryogenesis period, abnormal morphogenesis of the hepatobiliary may lead to syndromic BA or CBA. In our review, we summarized three susceptibility genes (*ADD3, GPC1, ARF6*) that participated in hepatobiliary development and be verified by animal disease models. As a progressive fibro-inflammatory disease, constant inflammation became BA's main pathological feature. Even after undergoing the KPE surgery, the inflammatory factors are constantly overexpressed. In the biopsy of BA liver, we can detect some changes in lymphocytes, including nuclear enlargement, loss of nuclear polarity, nuclear stratification, and vacuolated cytoplasm. Other inflammatory cells, including eosinophils, plasma cells, and macrophages, are also present (7). In recent years, the upregulation of proinflammatory genes, such as *MMP7, HMGB1, MIF*, and others, has been detected in much research. However, the high expression of these genes could be the cause or consequence of chronic cholestatic liver injury of BA. SNPs in some of these genes (*MIF, ITGB2, ICAM1*, and so on) provide strong evidence that these genes are the susceptibility gene of BA and their dysregulation leads to the inflammation phenotype of BA. Like inflammation, fibrosis is another main characteristic of BA and could be progressive even after KPE surgery. The activation of HSCs and EMT play a vital role in fibrosis progress. The participation of Growth factor signaling, the Fibro-genic signaling pathways and Chemokine pathways have all been found to be related with the activation of HSCs (92). In our review, we summarized three main genes and analyzed these functions in fibrosis of BA. For the first time, Andrew S Chu et al. proposed that the ciliary dysfunction and malformation were found in BA. Since 2012, much research has demonstrated that primary cilia may be the vital mechanism of BA. We summarized the variants in susceptibility genes and tried to demonstrate the relationship between the function of these genes and BA.

It should be noted that the genes listed above do not imply that they exert only one role in the pathogenesis of BA, as the majority of genes contribute to many biological processes. However, BA is not an isolate disease but a common pathology of various pathogenetic pathways. The 4 aspects are not clearly separable. The interplay between four aspects can eventually result in a particular phenotype of BA.

Thus, the identification and analyses of these gene expressions in BA will significantly improve our understanding of the pathogenesis for this condition. Future BA studies with larger cohorts and animal model research will be required to substantiate the roles and significance of these genes, such as *VEGFA, MIF, HMGB1, MAN1A2*, and other genes that may be involved in the pathogenesis of BA.

## Author Contributions

L-NW performed literature review, manuscript writing, and editing. L-YS and Z-JZ read, corrected, and supervised and coordinated all the work. All contributors read and approved the manuscript.

## Conflict of Interest

The authors declare that the research was conducted in the absence of any commercial or financial relationships that could be construed as a potential conflict of interest.

## Publisher's Note

All claims expressed in this article are solely those of the authors and do not necessarily represent those of their affiliated organizations, or those of the publisher, the editors and the reviewers. Any product that may be evaluated in this article, or claim that may be made by its manufacturer, is not guaranteed or endorsed by the publisher.
